# Design of the Dutch Obesity Intervention in Teenagers (NRG-DOiT): systematic development, implementation and evaluation of a school-based intervention aimed at the prevention of excessive weight gain in adolescents

**DOI:** 10.1186/1471-2458-6-304

**Published:** 2006-12-16

**Authors:** Amika S Singh, Marijke JM Chin A Paw, Stef PJ Kremers, Tommy LS Visscher, Johannes Brug, Willem van Mechelen

**Affiliations:** 1 EMGO Institute and Department of Public and Occupational Health, VU University Medical Center, Amsterdam, The Netherlands.; 2 Department of Health Education and Health Promotion, Universiteit Maastricht, Maastricht, The Netherlands.; 3 Institute for Health Sciences, Vrije Universiteit, Amsterdam, The Netherlands.; 4 Department of Public Health, Erasmus University Medical Centre Rotterdam, Rotterdam, The Netherlands.

## Abstract

**Background:**

Only limited data are available on the development, implementation, and evaluation processes of weight gain prevention programs in adolescents. To be able to learn from successes and failures of such interventions, integral written and published reports are needed.

**Methods:**

Applying the Intervention Mapping (IM) protocol, this paper describes the development, implementation, and evaluation of the Dutch Obesity Intervention in Teenagers (DOiT), a school-based intervention program aimed at the prevention of excessive weight gain.

The intervention focussed on the following health behaviours: (1) reduction of the consumption of sugar-sweetened beverages, (2) reduction of energy intake derived from snacks, (3) decrease of levels of sedentary behaviour, and (4) increase of levels of physical activity (i.e. active transport behaviour and sports participation).

The intervention program consisted of an individual classroom-based component (i.e. an educational program, covering 11 lessons of both biology and physical education classes), and an environmental component (i.e. encouraging and supporting changes at the school canteens, as well as offering additional physical education classes).

We evaluated the effectiveness of the intervention program using a randomised controlled trial design. We assessed the effects of the intervention on body composition (primary outcome measure), as well as on behaviour, behavioural determinants, and aerobic fitness (secondary outcome measures). Furthermore, we conducted a process evaluation.

**Discussion:**

The development of the DOiT-intervention resulted in a comprehensive school-based weight gain prevention program, tailored to the needs of Dutch adolescents from low socio-economic background.

## Background

Overweight and obesity in adolescence are two of the main predictors for obesity throughout the further life course [1-3]. The rising trends in excess weight among children and adolescents represent an emerging threat to public health [4], as overweight and obesity in adulthood are clearly linked to an increased risk for type 2 diabetes [5,6], cardiovascular disease [7,8], and some cancers [9]. The treatment of obesity has shown to be difficult and is often not successful in the longer term [10]. Population-based prevention of overweight and obesity may prove to be more efficient.

Dietary behaviour and physical activity patterns should both be targeted in the prevention of obesity. This may be achieved by inducing changes in personal and environmental mediators of such energy balance-related behaviours [11]. There is, however, little scientific evidence about successful population-based prevention programs in adolescents.

Several reviews on obesity prevention in children and adolescents have been produced during the last years [12-16]. Taken together, these reviews suggest that there is some evidence that prevention of obesity in children and adolescents is feasible. However, many intervention programs lack information on their development, exact content, and implementation [17], so that effective and ineffective components of these intervention programs cannot be determined. Hence, there is an obvious need for manuscripts that contain a detailed description of the development of the interventions under study, as well as their protocols for implementation and evaluation.

This paper describes the development, implementation, and evaluation design of the Dutch Obesity Intervention in Teenagers (NRG-DOiT), following the structure given by the Intervention Mapping protocol [18]. NRG-DOiT is part of a multi-disciplinary project group (NHF-NRG) that focuses on a better understanding of the obesity epidemic as well as the development of preventive measures [19].

## Methods

**Figure 1:**
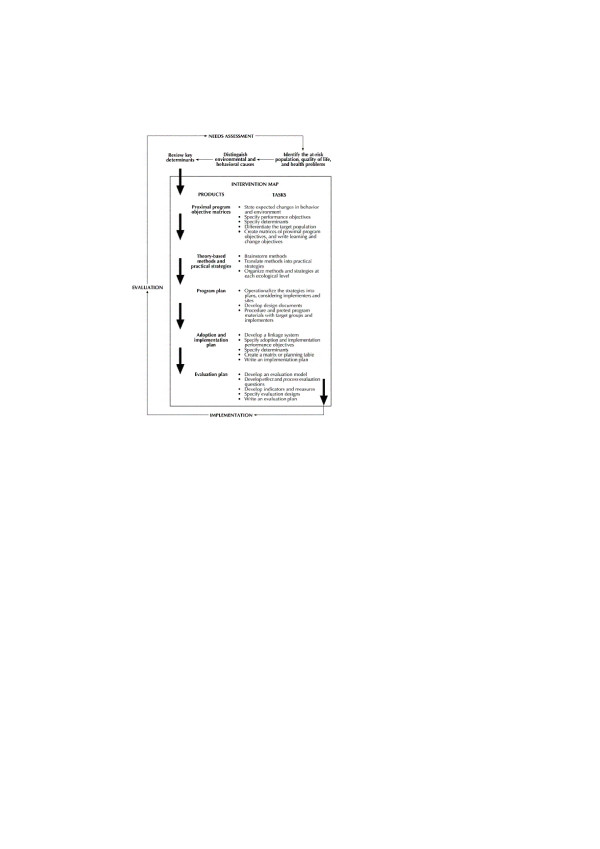
Intervention Mapping process. Source: Bartholomew et al. [18].

Intervention Mapping (IM) is a protocol that describes a stepwise process for theory and evidence-based development of health promotion programs [18] (figure 1). Concisely put, the three key input elements within IM are (1) a careful literature search for empirical findings, (2) the assessment and use of theory, and (3) the collection of new data.

The IM protocol consists of five steps: (1) the definition of program objectives, based on a thorough analyses of the health problem, (2) the selection of adequate theories and methods to realize behavioural change(s), (3) the design of the intervention program, as well as the selection, testing, and production of the intervention materials, (4) the development of a plan for the implementation, and (5) evaluation. Applying IM can be a complex and time-consuming process, but it ensures that each program objective is based on empirical evidence and theory. It also guarantees that intervention materials and activities are tailored to relevant characteristics of the target population [20], as well as to the abilities and opportunities or the program implementers and intermediaries.

Please note that the development of our intervention is based on the first edition of 'Intervention Mapping' [18], in which the 'needs assessment' is not an integral part of IM but precedes the IM process. In this paper, the needs assessment will therefore be described only briefly, the main focus being the description of the actual intervention background, contents, implementation plan, and evaluation design.

### Needs assessment

The starting point for the needs assessment was the fact that overweight and obesity are becoming the main determinants of preventable diseases in many countries, and the fact that overweight and obesity are often initiated in youth. Furthermore, during adolescence cognitive and behavioural competencies are developed, which are necessary to understand and act upon health and behavioural change instructions [21,22]. Therefore, health education programs aimed at adolescents may be successful in that they may lead to life-long healthy behavioural patterns, which in turn can positively influence both adolescent and adult health [2,23-26].

Our needs assessment included a structured analysis of the main risk behaviours related to weight management and becoming overweight in youth, resulting in the formulation of target behaviours on which the DOiT-intervention would be focussed. The methods of the needs assessment included an evaluation of reviews of scientific literature (if available), and relevant national and international reports on obesity and obesity-related topics. Furthermore, focus groups among adolescents were held, as well as interviews with teachers, and experts in the field of physical activity, dietary behaviour, and health promotion. During these focus groups and interviews, the risk behaviours identified in literature were discussed with the relevant key actors for the intervention. All interviews were conducted according to predefined protocols.

This procedure led to the identification of risk behaviours from both sides of the energy balance: energy intake and energy expenditure.

### Energy intake: sugar-sweetened beverages and snacks

The increase in the consumption of sugar-sweetened beverages [27,28] has been suggested to play an important role in excessive weight gain [29-31], and has shown to be associated with energy intake and body weight [32], and with weight gain and obesity [33]. Until now, only limited data are available on studies aimed at reducing the consumption of sugar-sweetened beverages in children and adolescents, but there is some evidence suggesting that avoiding the consumption of sugar-sweetened beverages can play an important role in obesity prevention [34].

Recent data show that as much as 30–35% of total energy intake of Dutch adolescents is derived from snacks and sugar-sweetened beverages [35]. Like sugar-sweetened beverages, the increasing frequency of snack consumption is positively correlated with total energy intake [36]. Considering that the problem of underreporting is very frequent in snack foods [37], the actual energy intake derived from snacks may even be higher than suggested.

Even though evidence of reducing adolescents' intake of high-sugar/high-fat products is limited, it seems obvious that the promotion of healthy dietary patterns should be part of obesity prevention programs [16].

### Energy Expenditure: sedentary behaviour and physical activity

Two important aspects of energy expenditure that contribute to excessive weight gain in adolescents are sedentary behaviour and declining levels of physical activity, both being behavioural and therefore modifiable [38].

TV viewing has been suggested to be one of the main contributors to the rising prevalence of childhood obesity: it replaces physical activity and it reduces resting metabolism. In addition, energy intake indirectly causes an increase of energy intake via advertising effects [39] and/or through providing increased opportunities for snacking and grazing behaviour [40-42]. Time spent watching television has shown to be significantly associated with prevalence of overweight in children and adolescents [41]. Children and adolescents spend an increasing amount of time on sedentary behaviours, like TV viewing and computer use. According to recent data, 42% of the Dutch adolescents aged 12–17 years spends 10–19 hours per week watching television, while 34% spends 20 hours or more [43]. Computer use has greatly increased during the last years. In 2002, Dutch adolescents spent seven hours per week on average using the computer (four hours using internet), whereas in 2004 it had increased to ten hours (seven hours using internet) [44].

Although physical activity is difficult to measure, especially in children, increasingly more data are available on duration and intensity of physical activity in children and adolescents. According to these data, levels of physical activity show a gradual decline with increasing age. This decrease is most marked at ages 13–16 [45,46].

Results of a recent review on the relationship between physical activity with overweight and obesity suggest that increased levels of physical activity and decreased levels of sedentary behaviour are protective against relative weight and fatness gains during adolescence [47].

**Figure 2:**
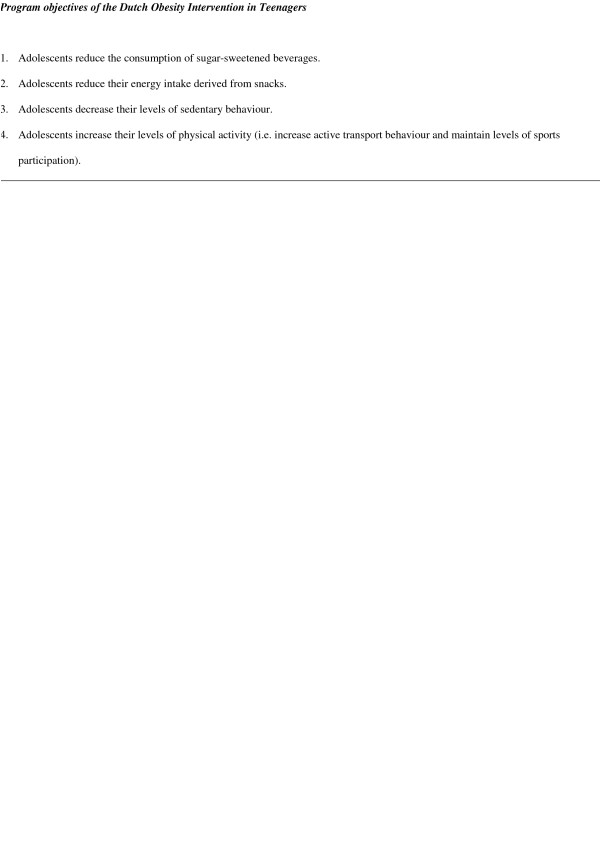
Example of performance objectives for program objective 1: 'Reduction of the consumption of sugar-sweetened beverages (SSBs)'.

Our needs assessment indicated that our intervention should address both dietary habits and physical activity patterns during adolescence, with the overall program objective being the prevention of excessive weight gain among Dutch adolescents. The health behaviours that should be targeted were translated into the formulation of the program objectives, presented in figure 2.

### Performance objectives

During this step of the IM process, the target group and the setting of the intervention was further specified to be able to tailor the intervention to the specific needs of the target group. In addition, performance objectives were specified, which were based on the program objectives identified during the needs assessment. The performance objectives clarified the exact behavioural 'performance(s)' expected from the target group. These performance objectives refer to changes in individual level behaviours, motivations, and abilities, as well as to environmental opportunities for such behaviours at the organisational and/or community level [20]. Finally, we developed matrices that combined the four identified risk behaviours and their hypothesized determinants. These matrices enabled the translation of the more general performance objectives into very specific intervention goals.

### General considerations

#### Person and environment

The poor success up to now of many interventions might be attributed to the fact that most of the interventions have aimed exclusively at behavioural change, using a traditional, individual-level approach. Egger and Swinburn [48] have instead proposed an ecological model that defines obesity as a net result of not only behavioural, biological, but also of environmental influences. Therefore, our intervention will address both individual as well as environmental level factors.

#### Targeting early adolescents from low-socio economic background

We decided to aim the intervention at adolescents attending prevocational secondary schools, in their first year (mean age 12–13 years). We selected this particular age group for several reasons. First, the onset of the decrease in physical activity-levels starts at age 13. Second, high levels of sedentary behaviour, unhealthy eating habits with regard to sugar-sweetened beverages, and high-sugar/high-fat content snacks can be observed in this age group. Since socio-economic status is inversely related to overweight [49,50], we decided to focus on adolescents with lower socio-economic and educational level.

#### The school setting

Schools can play a critical role in the prevention of overweight and obesity in children. With their existing organizational, social, and communication structures, they provide opportunities for health education and health enhancing environment on a regular basis [14,29].

In the Netherlands, adolescents attend secondary school at the prevocational educational level during four years. In general, they follow a basic educational program during the first two years, after which they choose a specific educational path, according to their desired occupational field. As the group composition is nearly the same during the first two years, this feature of the Dutch educational system makes both intervention and research in this age group very attractive.

At the environmental level, we defined school staff (teachers, headmasters, canteen employees) as the second target group, in order to accomplish environmental changes that facilitate behavioural changes among adolescents.

#### Specification of performance objectives

**Figure 3:**
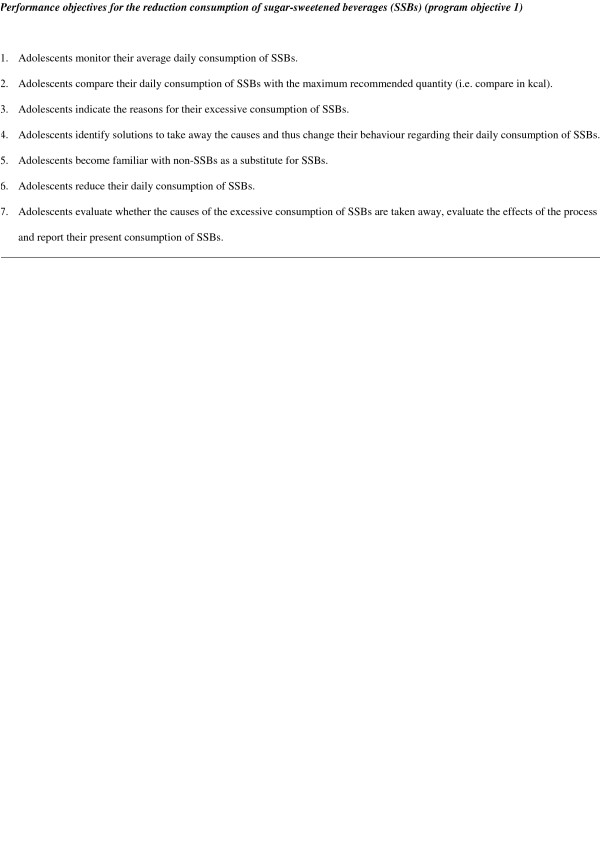
Program objectives.

Based on the self-regulation theory [51], seven performance objectives were specified for each of the program objectives at the individual level. By way of illustration, the seven performance objectives identified for the reduction of sugar-sweetened beverage (SSBs) consumption (program objective 1) are given in figure 3.

To be able to tailor the intervention to the specific needs of the target group, important and modifiable determinants of the target behaviours must be identified [52].

In Additional file 1 we present a matrix of performance objectives crossed with the individual and environmental determinants. These determinants are again based on a combination of an analysis of systematic reviews on determinants of energy balance-related behaviours [53-56], and personal interviews with teachers, parents, experts in the field of physical activity, dietary behaviour, and behavioural change.

### Theory-based methods and practical strategies

In this step of the Intervention Mapping process, theoretical methods and practical strategies that are likely to create the expected changes in the determinants were identified. Theoretical methods are general techniques or processes, derived from empirical evidence, mostly from behavioural and social science research [52], and describe the association between an intervention action and a change in behavioural determinants. Practical strategies are defined as techniques for the application of the theoretical methods. These strategies must be tailored to the specific needs of the target population and the setting where the intervention takes place. We identified relevant theoretical methods by literature review, investigation of existing programs [57], and in-depth interviews with teachers. Although underlying theoretical models and methods of behavioural change are rarely fully reported [17], Hardeman et al. [17] proposed a taxonomy of behaviour change methods that may assist individuals in developing interventions directed at weight gain prevention. Behaviour change methods, selected from this taxonomy, in order to change the performance objectives of NRG-DOiT were: (self) monitoring, self-evaluation, reward, increasing skills, goal setting, environmental changes, social encouragement, social support, information regarding behaviour and personalized messages.

In table 1 we present the theoretical methods that evolved from the selected health behavioural (change) theories. Practical strategies that were used to apply these methods within our intervention are illustrated using 'decreasing consumption of sugar-sweetened beverages' (program objective 1) as an example.

### Program plan

#### Program ideas

The name of the research project 'Dutch Obesity Intervention in Teenagers' led to the acronym 'DOiT', and became the central theme of the intervention program. 'DOiT' refers to the growth of behavioural self-determination, distinctive for the age of our target group. Close collaboration with professional designers and representatives of the target group representatives led to a 'fresh', 'cool', and 'not too childish', but 'more mature' logo (see Additional file 2) and appearance of the intervention materials.

Personal interviews with teachers and parents revealed that it was of utmost importance that words related to 'weight loss' or 'dieting' should be avoided in the intervention program. Hence, the main message of the intervention program consisted of 'maintaining energy balance', and extra attention was paid to distinct messages with regard to unhealthy dieting practices, such as anorexia nervosa or bulimia nervosa.

#### Intervention program and materials

Guided by tables 1 and 2 we first selected components from existing intervention programs that could fit into a school-based intervention program with a duration of one school-year (i.e. eight months, from October – May), tailored to the specific objectives of the DOiT-project. We adopted several ideas from a the 'Krachtvoer' program [57], an intervention promoting healthy eating among Dutch adolescents of the same age and socio-economic background as our target group. Suitable components were selected and integrated in cooperation with biology and physical education teachers, since they were supposed to implement the larger part of the program. If existing tools were not available, were not well enough tailored to the objective(s), were not accepted by the teachers, or their use was too costly, we either customized parts of already existing tools or developed new tools.

The DOiT-intervention program consisted of two components: an individual classroom-based intervention and an environmental intervention.

The individual classroom-based intervention consisted of an educational program, covering 11 lessons for the biology and physical education classes. Two 'schoolbooks' were developed, accompanied by specific worksheets for each lesson.

The first six lessons of the classroom intervention, named BALANCEiT (see Additional file 3), aimed at raising awareness and information processing with regard to energy balance-related behaviours. The adolescents monitored their own behaviour during three days, using a pocket-sized diary named CHECKiT (see Additional file 4), reported it back in the classroom, and received feedback from the teacher. Based on this monitoring process, the intervention program guided the adolescents in their choice which of the four risk behaviours they were going to change initially and helped to formulate implementation intentions [58]. Implementation intentions are not only formulated intentions of behaviours or behavioural changes to be accomplished, but they include a specification of circumstances wherein the behaviour is accomplished. The formulation of implementation intentions has shown to be effective in changing energy balance-related behaviours [59-61].

The second five lessons of the classroom intervention, named CHOOSEiT (see Additional file 5), aimed at facilitation of the choice to improve one of the identified risk behaviours. Assisted by the teachers and worksheets, adolescents identified their own risk behaviour(s), set personal goals, formulated implementation intentions, identified possible barriers/difficult situations, improved their self-efficacy, and evaluated change processes. Offering the adolescents the possibility of individual choice on how to maintain their energy balance, and not forcing them into one prescribed behavioural change, seems to be a potentially effective ingredient of our intervention [62]. To provide adolescents with individualized feedback and guide the adolescents through the change processes, a computer-tailored program was developed, which was based on existing materials [63], and accessible via the internet [64] or CD-rom. Computer-tailored education has shown to be more effective regarding dietary changes, especially with regard to the reduction of dietary fat, than general information [65].

To fit the DOiT-intervention optimally into the regular curriculum, the main objectives of the regular biology and physical education curricula, formulated by the Dutch Ministry of Education, Culture and Science, were taken into account.

The environmental part of the intervention consisted of a school-specific advice on the assortment of the school canteen, taking into account individual school characteristics and possibilities (for example distance to the nearest supermarket, policy with regard to permission to leave the school ground during breaks, or the relationship between the school canteen and the school board [in the Netherlands school canteens are run by either the school itself or outsourced to an independent entrepreneur]). Proposed change options were: offering smaller portion sizes (cans instead of bottles, normal size instead of king size); offering more 'healthy' products in the (products containing less fat, less sugar); or restricting access to vending machines (i.e. only after lunch break). In addition, we delivered posters for the school canteens, with 'traffic-light' suggestions for healthier choices. Foodstuffs were thus labelled as red ('Better do not'), yellow ('Only sometimes'), or green ('DOiT').

Furthermore, we encouraged the school board to offer additional physical activity options. We offered schools funding for two weekly hours of additional physical activity, under the following conditions: (1) The lessons should be supervised by a physical education teacher; (2) the lessons should fit within the school schedule (no break between the last official school lesson and the additional lesson physical activity); (3) a minimum number of twelve lessons should be taught between November 2003 and April 2004; (4) easy accessible activities, i.e. no specific knowledge or physical conditions necessary; (5) adolescents should be physically active during a major part of the lesson; (6) activities during the lessons should encourage adolescents to increase their leisure time physical activity as well.

#### Pre-testing of the materials

To explore whether the developed materials were acceptable for both adolescents and teachers, we pre-tested parts of the intervention materials during personal interviews with representatives of both groups. Pre-testing took place in schools that did not take part in the trial. Pre-testing revealed the need for low information density of the lessons (workbooks and worksheets), adapted language use to make the materials easy to understand, and not too much theory, but predominantly practical assignments.

#### Adoption and implementation plan

In this step of the IM process, a plan for the implementation of the intervention was developed. Performance objectives were formulated with regard to expected behavioural changes of implementers and adopters of the intervention program. To gain insight into facilitating factors and possible barriers regarding the implementation, teachers and school staff were interviewed at schools, willing and unwilling to adopt the program.

In the planning of the program, a time schedule for deliverance of the intervention program was formulated, taking into consideration extracurricular activities. Based on this schedule it was decided that the intervention should start October 2003 and end in May 2004, to ensure time wise a comparable implementation in all schools. All teachers received a manual describing the structure of each lesson and goals for the distinctive parts of the lessons.

The principle investigator indicated to be available for teachers via mail or telephone any time for possible questions on the intervention.

#### Evaluation plan

In this step of IM an evaluation plan and the corresponding evaluation measures were identified and developed, covering as much as possible the evaluation steps, as defined in the CONSORT statement [66,67], a checklist that intents to improve quality of reports of randomised controlled trials, by clarifying experimental processes.

We evaluated the intervention with regard to effects on body composition (primary outcome measure), behaviour, behavioural determinants, and aerobic fitness (secondary outcome measures). We also conducted a process evaluation to assess the reach, adoption, and implementation of the program, as well as conditions for program maintenance, such as appreciation of the project by students and school staff.

The effectiveness of the intervention program was evaluated using a cluster randomised controlled trial design, with measurements at baseline, after eight, twelve, and twenty months. The Medical Ethical Committee of the VU University Medical Center (Amsterdam, The Netherlands) approved the study protocol.

### Recruitment of the study population

#### Recruitment of schools

We recruited schools using the following three methods: (1) We sent written information about the study to approximately 400 schools, offering secondary prevocational education, located in the vicinity of maximal 150 kilometres from Amsterdam. One week later, all schools were approached by phone to ask for their willingness to participate; (2) in specialist journals for biology and physical education teachers, we placed advertisements to attract schools to participate; such schools could express their interest by mail, phone, or email; (3) we gave an oral presentation on a continuous education day for physical education teachers. Written information on study participation, including contact information, was handed out to those who were interested.

Biology teachers, physical education teachers, or first year coordinator were designated as possible contact persons. Schools that were interested (n = 20) were informed by means of an oral presentation on the demands of possible participation of the school in the DOiT-project. Inclusion criteria for the schools were: (1) being able to provide three classes to participate in the study, (2) willing to appoint a contact person for the duration of the trial, (3) willing not to change the curricula during the study period when assigned to the control group, and (4) being able to provide sufficient computer facilities in order to accomplish the lessons with the computer-tailored advice via internet or CD-rom.

If requested, we provided intervention materials to all first classes of the school and only charged the extra production-costs for the additional materials.

#### Recruitment of adolescents

The students and their parents received an information brochure about the study. There were no individual inclusion and exclusion criteria for study enrolment. As the aim of the intervention was to prevent excessive weight gain in all adolescents, and in order to avoid stigmatisation of the obese, children at all levels of body weight were eligible. Written informed consent was obtained from all students as well as their parents.

#### Randomisation procedure

Schools were randomly assigned to either the intervention or control group, using SPSS for a random selection of a sample. Randomisation took place at school level, and was stratified by urbanization grade (urban versus rural). Control schools were requested to maintain their regular curriculum for the duration of the project (two school years). Randomisation took place at the beginning of the school year, before baseline measurements took place, because intervention schools needed an adequate amount of time for the preparation of the classes.

#### Power calculation

The sample size calculation was based on differences between the experimental and control schools with regard to changes in body weight. A difference in weight gain of 0.5 kg between the intervention and control group after eight months was considered to be clinically relevant. Assuming α = 0.05, power = 0.90, and two-sided tests, 233 participants per group were needed to show a mean difference in weight of 0.5 kg (SD 1.5 kg) between the intervention and control group. To perform multi-level analyses and taking into account the cluster randomisation design and drop out (10–15%), a sample size between 500 and 600 subjects from 16 schools was required.

#### Measurements

**Figure 4:**
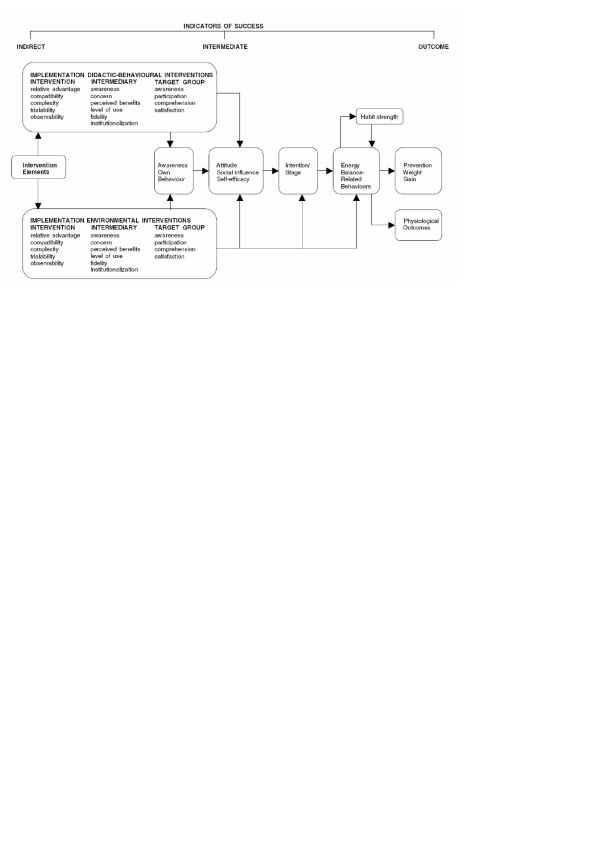
NHF-NRG intervention and evaluation model [19].

All measurements were completed at baseline, after eight, twelve, and twenty months. Choice of measurements was based on the NHF-NRG evaluation model (figure 4).

To minimize seasonal influences, all measurements were performed within a six-week period in all schools. Measurements were completed according to a standardized protocol by a trained research team. The research assistants were also involved in the organization of the measurements, and were therefore not be blinded to group assignment. Each measurement period the research team consisted of two research assistants, who performed all anthropometric measurements (one for female adolescents, one for male adolescents), two master students who assisted the research assistants (e.g. writing down the measurement results), and the principle researcher, who performed the shuttle-run test.

Before the measurements, the contact person was asked to designate two lessons physical education per class (approximately 30 students) to complete the anthropometric measures and the shuttle run test, and two other lessons for the completion of the questionnaires as a classroom activity. During the physical measurements, at least one teacher (preferably the physical education teacher) had to be present. After completion of each measurement period adolescents received a small incentive (key ring, mugs, etc).

#### Physical measurements

At the start of the measurements, the procedure of the physical measurements was explained to the students. Then, students were divided into three groups of about ten students. While the first group was measured (body weight, height, skin folds, waist and hip circumference), the second group performed the shuttle run test, and the third group was waiting. When the first two groups had completed their measurements, the groups transferred to the next measurement section, until every student had completed all measurements. Optimal privacy was assured during the anthropometric measurements by measuring one adolescent at a time in a separate room.

#### Body height and body weight

Body height was measured and recorded with an accuracy of 1 mm with a portable stadiometer. We assembled a level to the stadiometer to ensure that the head position was standardized during the measurement. Body weight was measured and recorded within 0.1 kg with a calibrated electronic flat scale, levelled after each placement.

#### Skin folds, waist and hip circumference

Skin folds (m.triceps, m.biceps, m.suprailiacalis, m.subscapularis) were measured at the right side of the body to the nearest 0.2 mm [68]. We determined skin fold thickness by averaging two measurements. If the two measurements differed by more than 1 mm, a third measurement was performed, and skin fold thickness was taken as the average over the three measurements. Waist and hip circumference were measured with a flexible band to an accuracy of 0.5 cm.

Before each measurement period, intra-rater and inter-rater reliability for all four skin folds were determined. Values for intra-rater reliability (ICC) varied between 0.82 and 0.95. Values for inter-rater reliability (ICC) varied between 0.88 and 0.99.

#### Physical fitness

To assess aerobic fitness, a group-administered shuttle run test (SRT) was conducted [69]. Due to limited size of the sports hall at several schools we changed the distance for each shuttle from 20 to 18 meters at all schools. We used the protocol of the Eurofit test [70]. The SRT consists of a number of stages, each lasting one minute, paced by beeps at set intervals. Participants ran the 18-meter course back and forth, starting at a speed of 7.2 km/h. As the test proceeds, the interval between each successive beep reduces, forcing the student to increase running speed. The SRT test has been shown to be a reliable and valid field test to estimate maximal oxygen uptake (VO_2_max) in 8–19 year old children [69,71]. To increase motivation the SR test was preformed with music. Each measurement period the same music were used.

### Measurements on behavioural outcome measures

#### Objective measure of physical activity

In a sub sample of the study population (five schools, one class each) physical activity levels were assessed objectively using Manufacturing Technology Inc. (MTI) accelerometers (model 7164). The MTI is a valid [72-74] and reliable [75] instrument to measure vertical displacement of the human body.

Depending on the number of participants on each school, 15 to 25 adolescents were asked to wear the accelerometer for six consecutive days, since Trost et al. [76] found that a minimum of four to five days of monitoring was necessary to establish a reliability of at least 0.80. The adolescents were asked to wear the accelerometer during all waking hours, and to remove it during swimming, bathing and showering.

The participants as well as their parents received a letter with uniform instructions on how, when and where the accelerometer had to be worn. The accelerometer was secured by a waist belt and worn on the right side of the hip. To overcome the problem of inter-instrument differences, we aimed to provide each participant with the same accelerometer during all four measurement periods.

#### Questionnaires

Students were requested to complete the questionnaires in the classroom. Before the questionnaires were handed out, a member of the DOiT-team shortly explained the procedure for the completion of the questionnaire. A teacher and/or a member of the DOiT-team supervised the completion of the questionnaires and took care of collecting the completed questionnaires. On average the adolescents required 60 minutes to complete the questionnaire.

#### - Behaviour

Questionnaires contained frequency questions regarding the four identified risk behaviours: (1) consumption of sugar-sweetened beverages, (2) consumption of high-sugar/high-fat content snacks, (3) sedentary behaviour (watching television and using the computer), (4) low levels of physical activity (i.e. active transport to school and participation in physical activity and sports).

The questionnaire was based on other validated questionnaires for assessing dietary intake, physical activity, behaviour-specific cognitions, and habit strength in adolescent populations [77-79]. We adjusted the questionnaires according to our study population.

The structure of the questionnaire was equal for all risk behaviours. For example, adolescents had to indicate how many days a week they consumed sugar-sweetened beverages, and the amount/number of servings of sugar-sweetened beverages they usually consumed on these days, thinking of the last week. Frequency and quantity were multiplied to obtain estimates of mean daily consumption.

#### - Motivational determinants of behaviour

Questions on personal and social environmental determinants of each of the risk behaviours included questions on attitude, subjective norm, perceived behavioural control, personal barriers, intention and habit-strength. The determinant variables were based on the EnRGframework [80], an integrative framework that applies insights from Dual-Process Theory [81], the ANGELO model [82], the Theory of Planned Behaviour [83], and habit theory [84]. Most variables were measured on bipolar five-point Likert scales.

The self-administered questionnaire was pre-tested for clarity and length, by means of cognitive interviewing of four adolescents from a school not participating in the study [85].

#### Statistical analyses

To evaluate the effects of the intervention, multilevel analysis was used. Using this technique, regression coefficients can be adjusted for the clustering of observations within one school and/or class. We defined three levels in our multi-level analysis: 1) student, 2) class, and 3) school. Linear and logistic models were used to examine the effect of the intervention on each of the outcome values. All analyses were performed according to the intention-to-treat-principle, that means that we did not impute values for missing data [86].

#### Process evaluation

Process evaluation was based on the Diffusion of Innovations Theory [87], and involved data gathering among pupils, teachers, and members of the school boards.

#### Teachers and members of the school board the intervention program

All teachers and members of the board, who were involved in the implementation of the DOiT-program, were asked to participate in a semi-structured interview (questionnaire) during and after the intervention period. The questionnaire mostly consisted of structured questions, measured on a bipolar five-point Likert scale (e.g. much better, better, not better/not worse, worse, much worse). A couple of open-ended questions were added. The questionnaire was based on already existing questionnaires, used to evaluate interventions aimed at enhancing physical activity applied by general practitioners [88,89], and consisted of 31 questions. The questionnaire contained questions on the overall impression of the DOiT-project, contentment with the communication with the DOiT team, and time spent on implementing the DOiT-program. The teachers' manual, educational appliances, the implementation of the intervention, and barriers for correct implementation were also evaluated. Finally, the intention and opportunities for implementing the DOiT-program in the future were discussed.

Additionally, we aimed to visit minimally one lesson of the classroom part of the intervention for a structured observation.

For the additional lessons physical activity, teachers were requested to register the presence of the adolescents. Forms were handed out to register activities, and teachers were asked to send the forms back monthly. We aimed to visit minimally one lesson additional physical activity at each school, checking whether the required conditions were fulfilled (by means of a form).

At the end of the intervention, all teachers physical education involved in the supervision and organization of the additional lessons physical activity were sent a short evaluation questionnaire.

#### Adolescents

During the second measurement (after eight months) questions on the lessons about healthy food, physical activity behaviour, sedentariness, changes in the assortment of the school canteen, and additional lessons physical activity were added to the questionnaire. During the last lessons of the intervention, a short questionnaire was handed out to the students of the experimental schools, evaluating the intervention program shortly (ten questions on content and attractiveness of the lessons, including intervention materials).

## Discussion

We applied the IM protocol for the development of an intervention aiming at the prevention of excessive weight gain among Dutch adolescents. We perceived the use of the IM protocol as a useful tool that has guided us through the process of the development of our intervention program. Since application of the IM protocol, as described by Bartholomew et al. [18], demands more time than was available within the DOiT-project, we decided to take a short-cut in the IM process. Following examples of other research groups that had applied the IM protocol before [57,90], we modified the original protocol by restricting the amount and complexity of matrices in steps 1 and 2. Although we perceived the development of our intervention according to the IM as a complex and time-consuming process, we believe that the application of theory will improve the likelihood of effectiveness of interventions [91], and the several feedback loops in the process assured that the program matched the needs of our target population.

In conclusion, the development of the DOiT-intervention according to the IM protocol resulted in a comprehensive school-based weight gain prevention program, specifically tailored to the needs of Dutch adolescents. To determine the effectiveness of our program, we examined body composition, behaviour and behavioural determinants, and aerobic fitness. Furthermore, a process evaluation was conducted to gain insight in effective and ineffective parts of the intervention.

The results of the analyses will help to gain more insight school-based prevention of overweight and obesity in adolescents. Results of the intervention will be available in 2007.

## Competing interests

The author(s) declare that they have no competing interests.

## Authors' contributions

AS, MC, SK, TV, JB, and WvM provided support in the design of the study and contributed intellectual input into the main ideas of this paper. AS designed and coordinated the implementation of the intervention. She supervised data-collection, analysed data, and drafted the manuscript. MC, SK, and TV provided support during the development of the intervention. All authors contributed to the further writing of the manuscript. MC, JB, and WvM obtained financial support. AS will act as guarantor of the paper. All authors read and approved the final manuscript.

## Pre-publication history

The pre-publication history for this paper can be accessed here:



## Supplementary Material

Additional file 1 Performance objectives related to changes in behavioural and environmental determinants, with regard to consumption of sugar-sweetened beverages (SSBs) Click here for file

Additional file 2 Logo Dutch Obesity Intervention in Teenagers (DOiT) Click here for file

Additional file 3 Intervention material individual intervention, schoolbook 'BALANCEiT' Click here for file

Additional file 4 Intervention material individual intervention, pocket-sized diary 'CHECKiT' Click here for file

Additional file 5 Intervention material individual intervention, schoolbook 'CHOOSEiT' Click here for file

## Figures and Tables

**Table 1:** Theoretical methods matched with practical strategies, identified for the DOiT-intervention program. Example: determinants of reduction consumption of sugar-sweetened beverages (SSBs) (program objective 1)

**Determinant**	**Methods from theory**	**Strategy**	**Tools/Materials**
Knowledge	Passive learning/providing information	Providing written and verbal information	Information in print materials (workbook/worksheets) during school lessons on label reading health consequences of excessive consumption SSBs Videotape on clandestine advertising Posters in school canteen
	Active processing of information	Evaluating understanding	Knowledge tests (quizzes: DRINKiT) Cut and paste worksheets regarding SSB Role model stories Group discussions Questionnaires (TESTiT) Test recipes (workbook/worksheet) Invent own recipes (website)
Awareness	Self-monitoring and feedback	Monitoring of own behaviour (social) comparison	Pocket-size diary (CHECKiT, 3 days) Worksheets Computer-tailored advice
Skills	Guided practice	Skills training (label reading and taste) Provide feedback	Practice label reading in groups Worksheets
Social support	Social modelling/peer modelling/social comparison	Providing information	Workbook/worksheets/computer tailored advice (questionnaires, website or CD-rom) Role play Group discussions
Habit	Breaking automatic stimulus-response relations, rising awareness of habitual behaviour	Changing the environment/point-of-decision prompts	Posters near food access points/changing canteen assortment
Self efficacy	Goal setting	Formulation of implementation intentions Individualized feedback	Worksheets to help exact planning goals (How? When? Where? With whom?) Role model stories Discussion barriers/difficult situations, possible solutions Computer tailored advice (website or CD-rom)
	Reinforcement	Providing feedback Evaluation of change processes Analyses difficult situations	Computer tailored advice (website or CD-rom) Worksheets Group discussions
	Association of attitude object with other positive stimuli		Role model stories Group discussions
	Environmental changes	Facilitation of healthy behaviours	Changes in school canteen assortment Posters in school canteen
