# Supplementary material for: Design of the Dutch Obesity Intervention in Teenagers (NRG-DOiT): systematic development, implementation and evaluation of a school-based intervention aimed at the prevention of excessive weight gain in adolescents
Source: BMC Public Health. 2006 Dec 16;6:304. doi: 10.1186/1471-2458-6-304 (PMC1769372; doi:10.1186/1471-2458-6-304)
Supplement: Additional file 1 — Performance objectives related to changes in behavioural and environmental determinants, with regard to consumption of sugar-sweetened beverages (SSBs) [file 1471-2458-6-304-S1.doc]

**Additional file 1.** Performance objectives related to changes in behavioural and environmental determinants, with regard to consumption of sugar-sweetened beverages (SSBs)

| **Adolescents (A) reduce the consumption SSBs.** | **Individual determinants** | | | | **Social-environmental determinants** |  |
| --- | --- | --- | --- | --- | --- | --- |
| Target group | Adolescents (A) | | | | Peers/parents (P) | Employees responsible for school canteen (SC) |
| subbehaviours | Knowledge | Awareness / Attitude | Skills | Self-efficacy | Social support/social pressure | Availability/accessibility |
| 1. A monitor their average daily consumption of SSBs.  2. A compare their daily consumption of SSBs with the maximum recommended quantity. | A know/learn how to identify SSBs (correct label reading).  A know what the recommended amount of SSBs is. | A become aware of the differences between SSBs en non-SSBs.  A become aware of their average daily consumption of SSBs.  A report the availability of SSBs in their horme environment. | A note down how much and when SSBs are consumed.  A compare their daily consumption of SSBs with the recommended quantity of SSBs.  A learn to compare and describe the differences in taste between SSBs and non-SSBs. | A feel confident about reading the information on the packing of SSBs en non-SSBs.  A feel confident about registering their daily consumption of SSBs. |  | SC know the differences between SSBs and non-SSBs.  SC are aware of the composition of the assortment they offer with regard to SSBs and non-SSBs. |
| 3. A indicate what the reasons are for their excessive consumption of SSBs.  4. A identify solutions to take away the causes and thus change their behaviour regarding to their daily consumption of SSBs.  5. A become familiar with non-SSBs as substitute for SSBs. | A learn about the negative health consequences of excessive consumption of SSBs.  A learn about the positive health consequences of healthier choices. | A become aware of difficult situations / barriers.  A feel positive about non-SSBs as possible substitutes for SSBs.  A become aware of the negative health consequences of excessive consumption of SSBs/positive health consequences of healthier choice.  A become aware in what kind of situations they consume SSBs and what influences their consumption behaviour (advertisement/peers/parents/habit). | A can describe the reasons for their excessive consumption of SSBs.  A learn to compare and describe nutritional and taste differences between SSBs and non-SSBs.  A are able to identify possible substitutes for SSBs.  A can name relevant barriers / difficult situations that can be expected and give possible solutions.  A gain insight that their behaviour often depends on situational cues.  A learn to set feasible goals (characterized by small behavioural changes). | A feel confident about being able to influence their behaviour by taking away (some of) the identified causes.  A feel confident to try and compare SSBs with non-SSBs. | P become aware of the fact their children want to reduce their consumption of SSBs.  Parents are aware of possible substitutes for SSBs and make those available at home. | SC become aware of advertisements for SSBS and non-SSBs.  SC support availability of non-SSBs and restrict availability of SSBS (i.e. time SSBs are available, decrease portion sizes of SSBs).  SC highlight differences between SSBs and non-SSBs. |
| 6. A reduce their daily consumption of SSBs.  7. A evaluate if the causes of the excessive consumption of SSBs are taken away, evaluate the effects of the process and report their present consumption of SSBs. | A know how to reduce their daily consumption of SSBs (i.e. smaller portion sizes, choosing substitutes). |  | A buy/ask for non-SSBs in different situations.  A demonstrate skills to find ways to handle difficult situations / barriers.  A can evaluate eventual behavioural changes with regard to SSBs (monitoring) and causes for success or failure thereof. | A feel confident about being able to act according to the formulated implementation intentions in difficult situations.  A feel confident to choose substitutes for SSBs. | P help their children in difficult situations to replace SSBs by non-SSBs, and so reduce their consumption of SSBs. |  |
