# Supplementary figures and images for: Design of the Dutch Obesity Intervention in Teenagers (NRG-DOiT): systematic development, implementation and evaluation of a school-based intervention aimed at the prevention of excessive weight gain in adolescents
Source: BMC Public Health. 2006 Dec 16;6:304. doi: 10.1186/1471-2458-6-304 (PMC1769372; doi:10.1186/1471-2458-6-304)

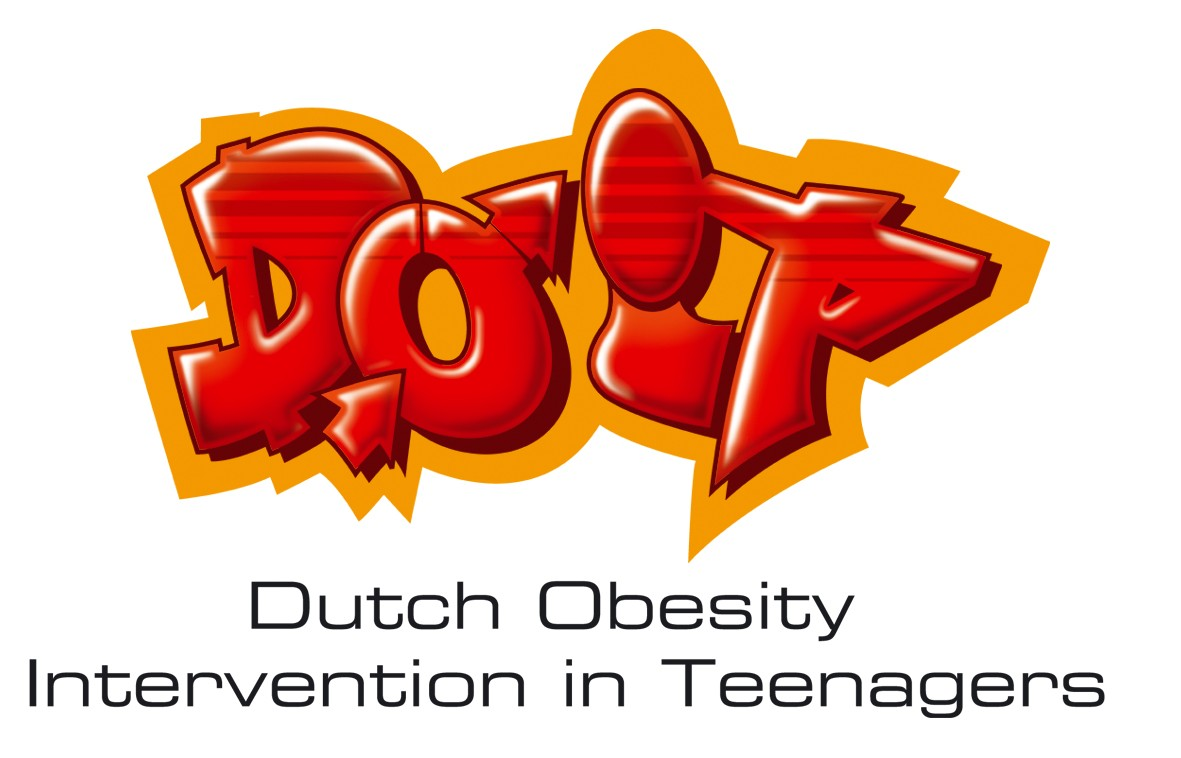

Supplement: Additional file 2 — Logo Dutch Obesity Intervention in Teenagers (DOiT) [file 1471-2458-6-304-S2.doc]

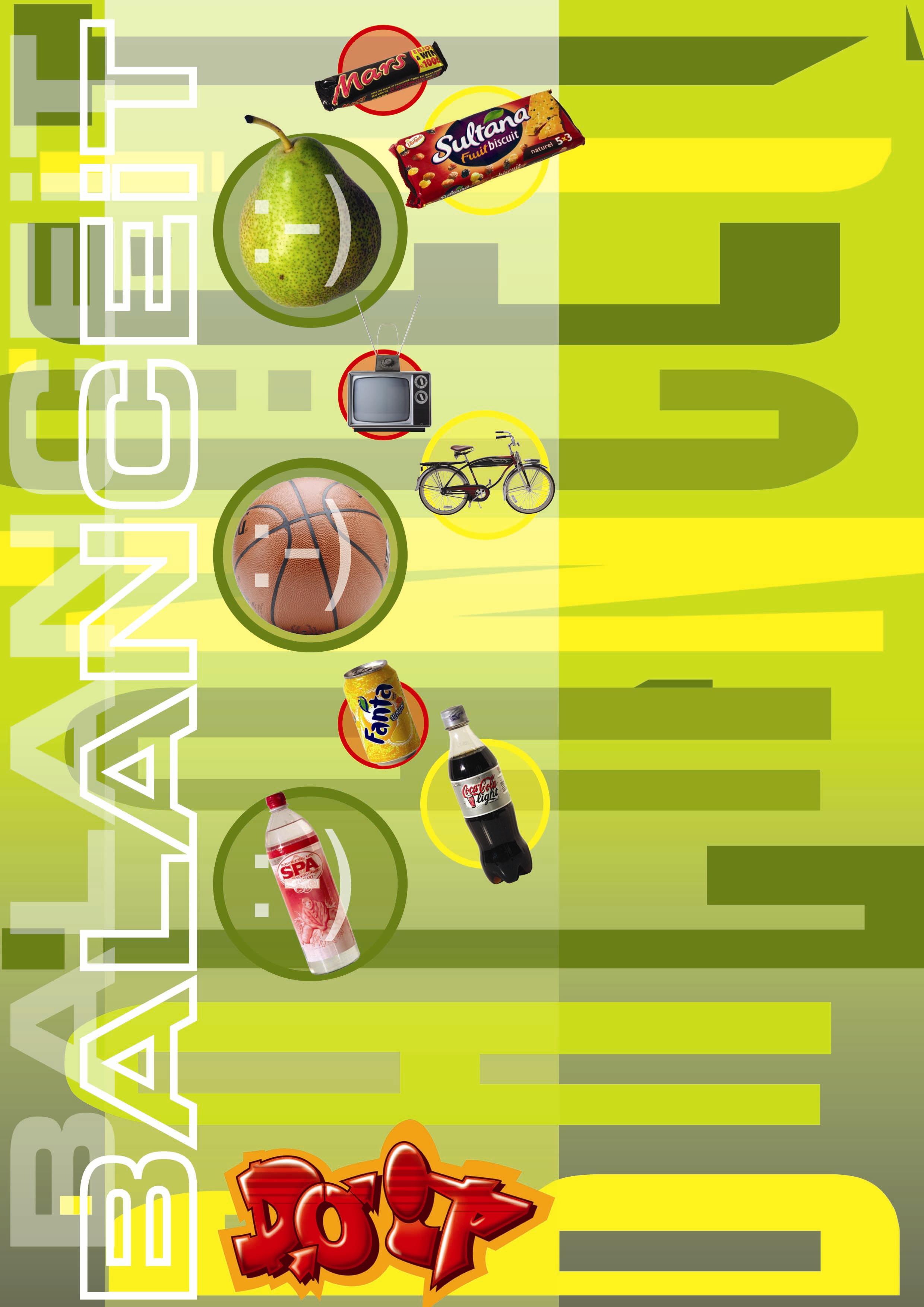

Supplement: Additional file 3 — Intervention material individual intervention, schoolbook 'BALANCEiT' [file 1471-2458-6-304-S3.doc]

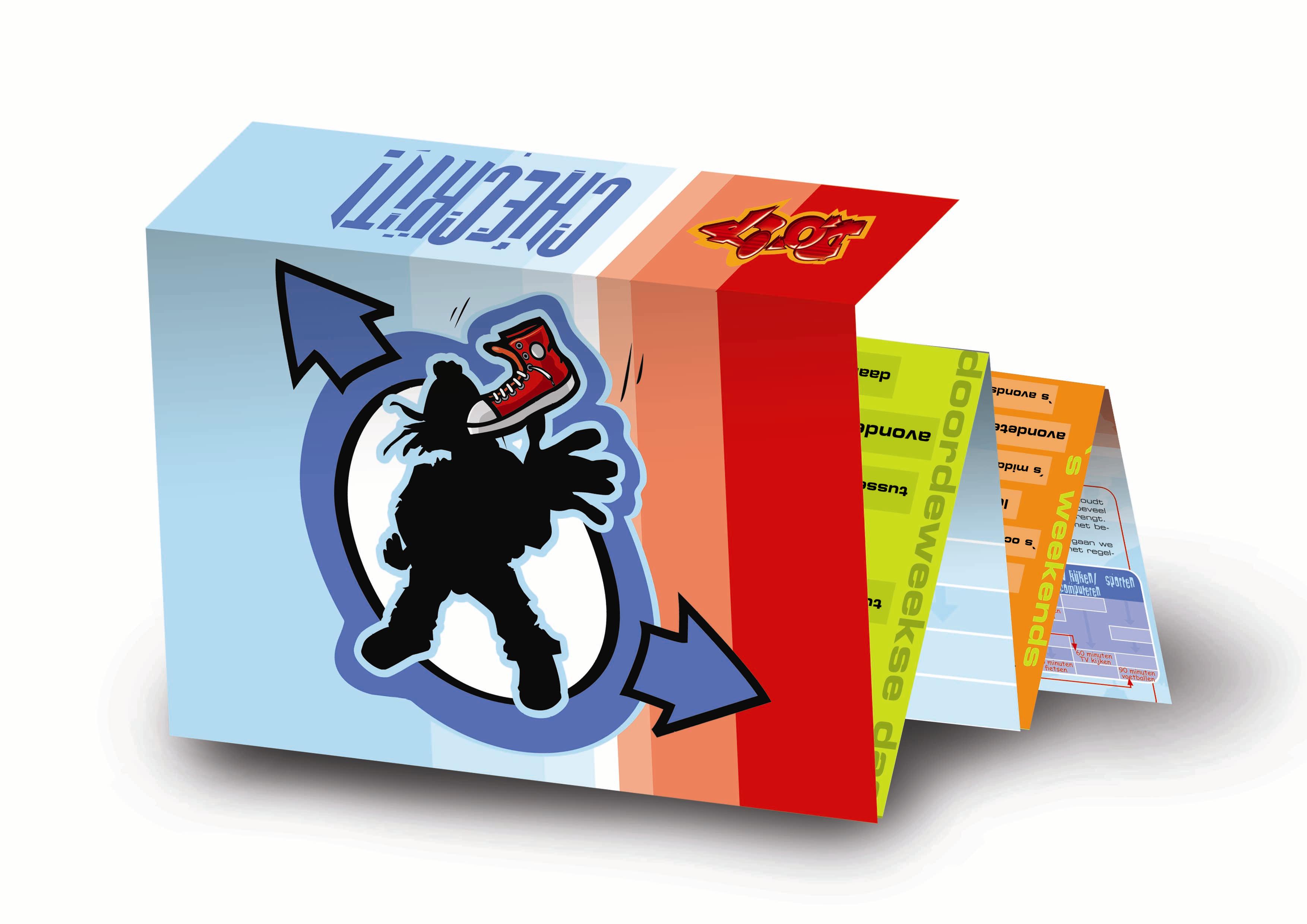

Supplement: Additional file 4 — Intervention material individual intervention, pocket-sized diary 'CHECKiT' [file 1471-2458-6-304-S4.doc]

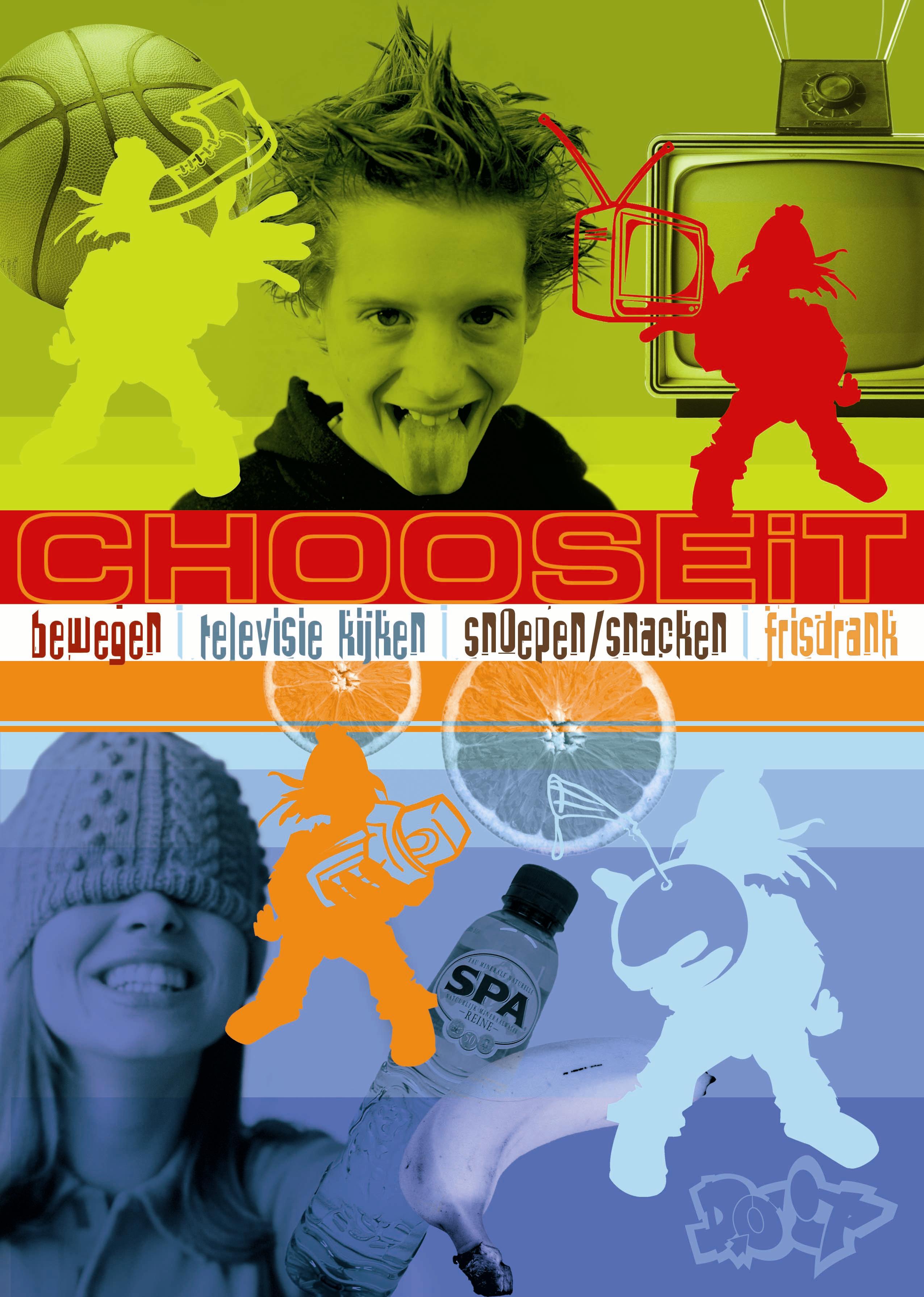

Supplement: Additional file 5 — Intervention material individual intervention, schoolbook 'CHOOSEiT' [file 1471-2458-6-304-S5.doc]
